# Supplementary figures and images for: Don’t judge a book or health app by its cover: User ratings and downloads are not linked to quality
Source: PLoS One. 2024 Mar 4;19(3):e0298977. doi: 10.1371/journal.pone.0298977 (PMC10911617; doi:10.1371/journal.pone.0298977)

## Appendix 1


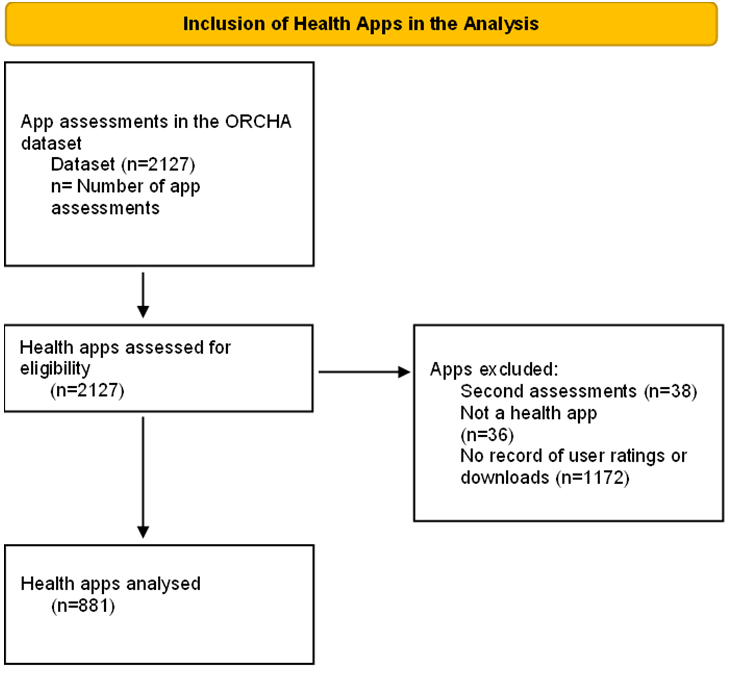


**Appendix 1 Fig 1**: Health app selection for the analysis

Supplement: S1 Appendix — (DOCX) [file pone.0298977.s001.docx]
