# Supplementary material for: Don’t judge a book or health app by its cover: User ratings and downloads are not linked to quality
Source: PLoS One. 2024 Mar 4;19(3):e0298977. doi: 10.1371/journal.pone.0298977 (PMC10911617; doi:10.1371/journal.pone.0298977)
Supplement: S2 Appendix — (DOCX) [file pone.0298977.s002.docx]

## Appendix 2

Appendix 2 Fig 1 depicts boxplots of each score against user rating. Appendix 2 Figs 2-5 depict boxplots of each score against download level. Above each box plot is a number indicating the sample size. Sample size (depicted in the Figs) varied from 1 to 177, this is because download level ‘1,2,3 and 19’ has a sample size of 0 and was not included in the Fig.


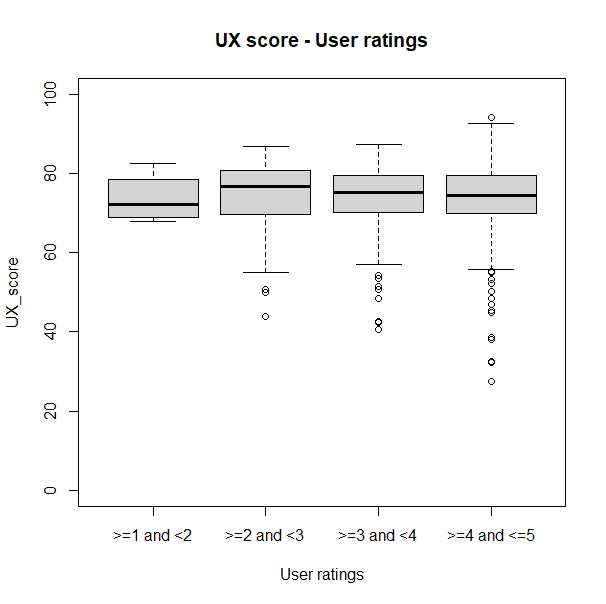

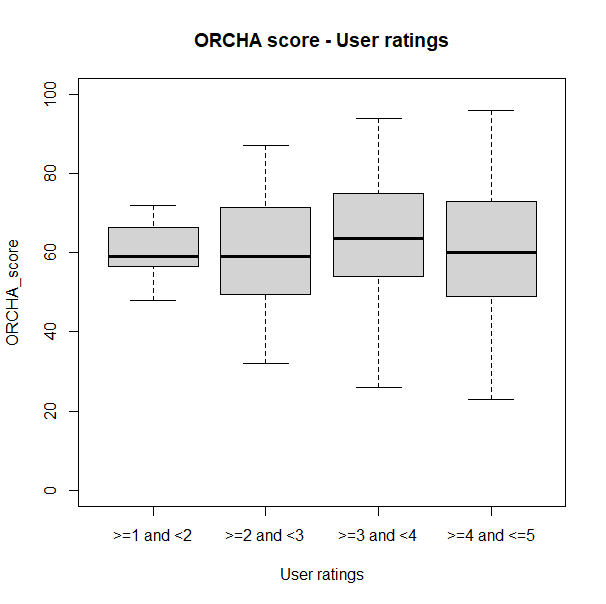

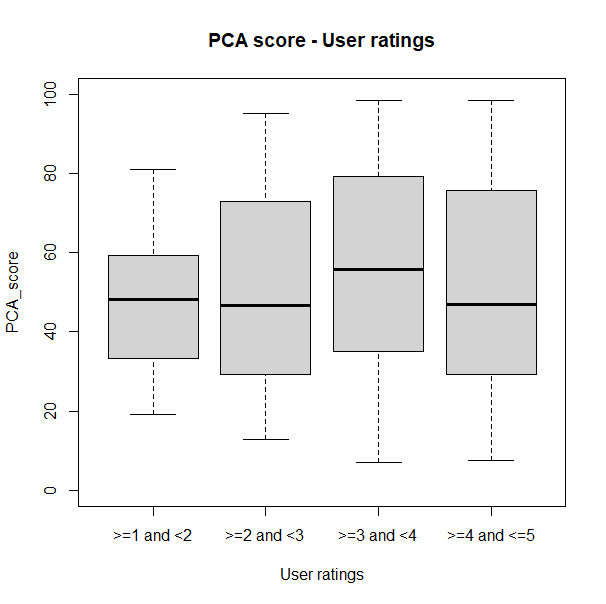

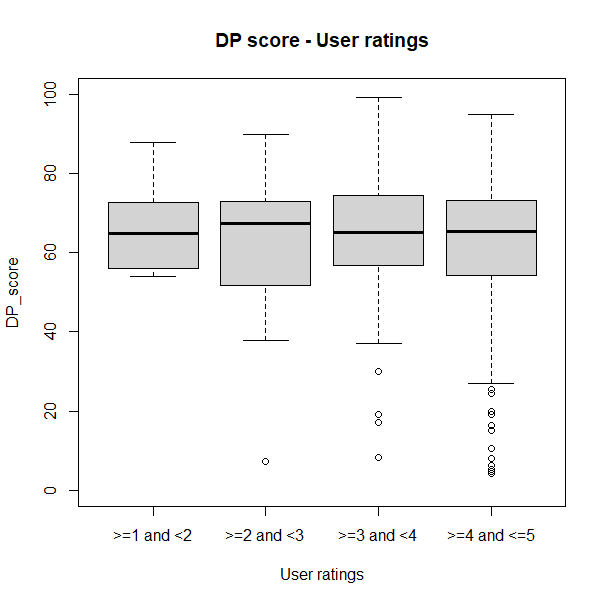


8

43

222

608

8

43

222

608

8

43

222

608

8

43

222

608

**a) b)**

**c) d)**

**Appendix 2 Fig 1:** Numbers above boxplots are sample sizes. a) ORCHA score against user ratings boxplots. b) UX score against user ratings boxplots. c) PCA score against user ratings boxplots. d) DP score against user ratings boxplots.


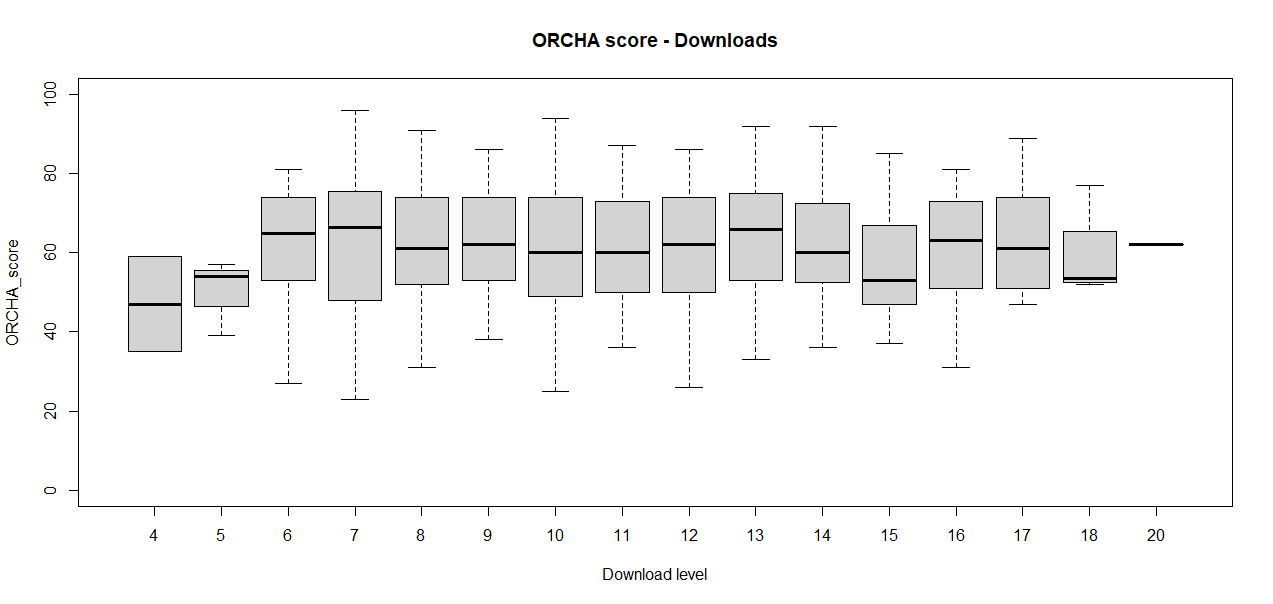


2

3

30

28

139

70

177

70

134

61

72

31

53

6

4

1

**Appendix 2 Fig 2:** ORCHA score against download level boxplots.


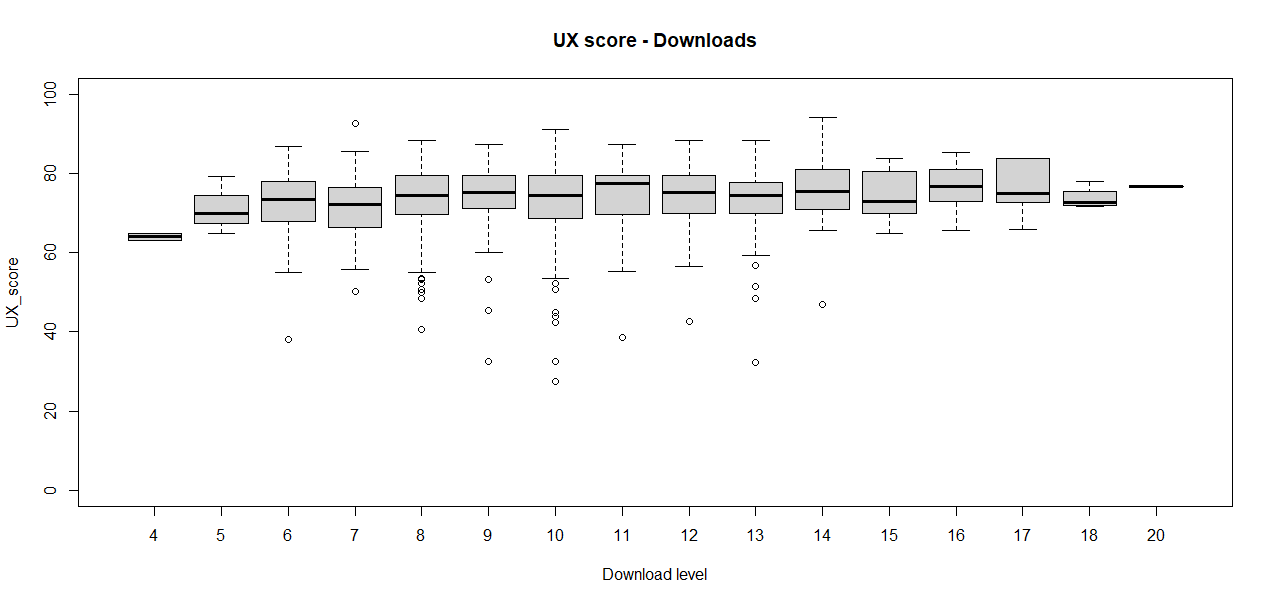


2

3

30

28

139

70

177

70

134

61

72

31

53

6

4

1

**Appendix 2 Fig 3:** UX score against download level boxplots.


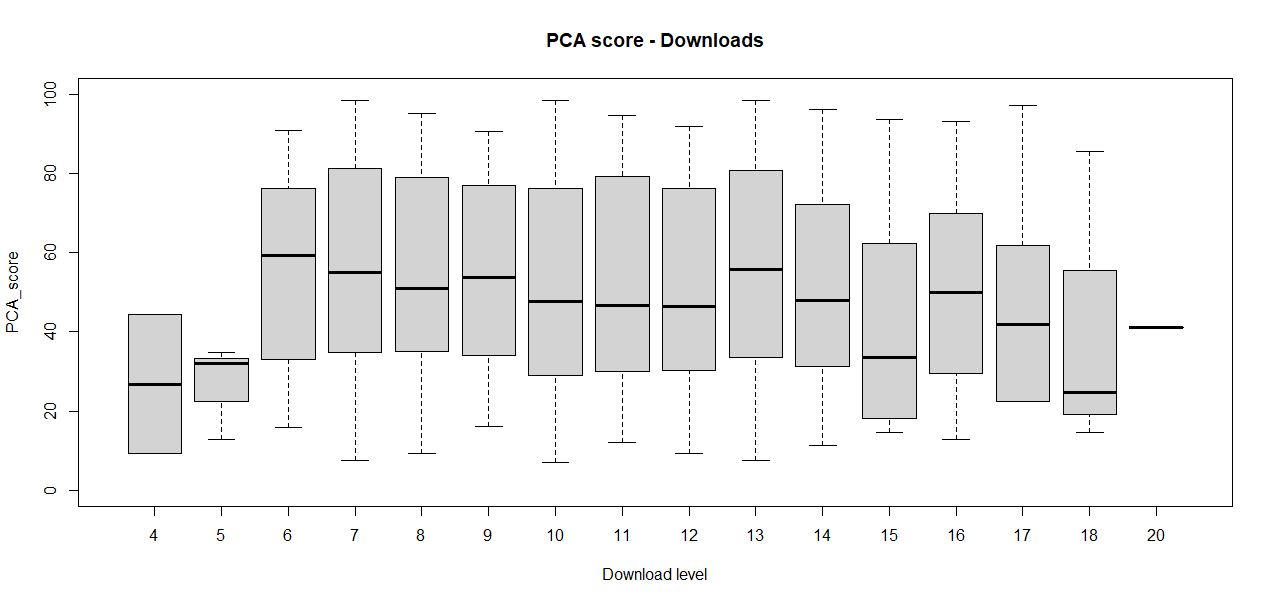


2

3

30

28

139

70

177

70

134

61

72

31

53

6

4

1

**Appendix 2 Fig 4:** PCA score against download level boxplots.


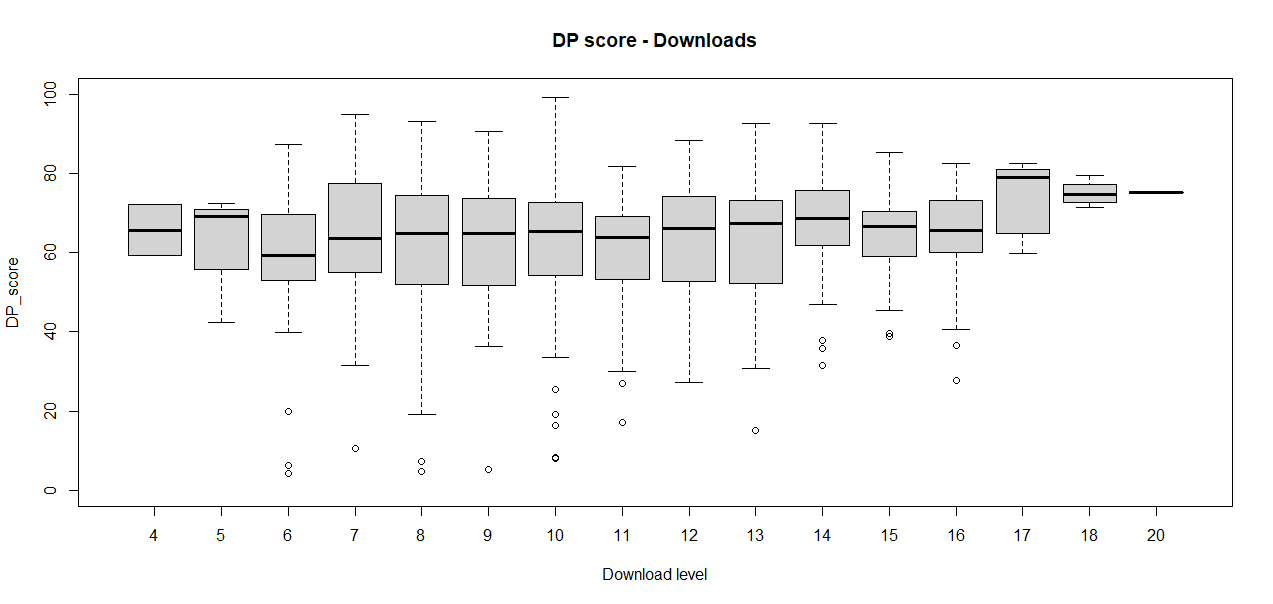


2

3

30

28

139

70

177

70

134

61

72

31

53

6

4

1

**Appendix 2 Fig 5:** DP score against download level boxplots.
