# Supplementary material for: Don’t judge a book or health app by its cover: User ratings and downloads are not linked to quality
Source: PLoS One. 2024 Mar 4;19(3):e0298977. doi: 10.1371/journal.pone.0298977 (PMC10911617; doi:10.1371/journal.pone.0298977)
Supplement: Attachment — Submitted filename: Reviewer response.docx [file pone.0298977.s004.docx]

Dear Editor and Reviewers,

Thank you for the feedback and the opportunity to improve our manuscript. We have now addressed reviewers’ comments. Below is our point-by-point response to the comments.

Reviewer #1: Thank you for sharing this manuscript.

I found your conclusions sound and a valuable addition to the literature.

I do think you need to make some adjustments to your statistical analysis. Primarily, you perform several tests of correlation and several MLRs, and therefore you should be correcting your p-values for multiple hypothesis testing. As this will only serve to make these values less statistically significant, this will not change the conclusions of your study (and indeed will likely make some of the statistically significant correlations no longer significant).

Reviewer 1 response A1: We acknowledge that the p-values should be corrected for multiple hypothesis testing. We have now revised the analysis and included Bonferroni corrected alpha values.

Reviewer #2: Thank you for the opportunity to review your manuscript comparing user ratings to objective assessments of mobile apps for health. Overall: Some awkward sentence structures and minor grammatical errors that can easily be corrected.

Introduction: Page 4, lines 88-90: In the sentence: “Therefore, if not sufficiently informed, user’s app choices can result in foregone health benefits if ineffective apps are chosen and/or can pose significant risks to user’s health and privacy.” I wonder if you mean poor health benefits instead of foregone.

Reviewer 2 response A1: We have now reworded the sentence to “Therefore, if not sufficiently informed, user’s app choices can result in poor health benefits if ineffective apps are chosen and/or pose significant risks to user’s health and privacy.”

Methods: Could you please give a bit more detail about how you determined the download levels from the ranges? It may also be useful to your readers to have a brief explanation about why you chose the analyses described.

Reviewer 2 response A2: The following paragraphs were modified in the manuscript.

“Data was accessed and analysed between ‎July and December 2022. We carried out secondary data analyses of this ORCHA dataset, using R studio and R programming language. Spearman correlations were used to examine how correlated ORHCA, UX, PCA and DP scores are with user ratings (a 1-5 rating) and number of downloads. The number of downloads variable was converted into download levels, as only download ranges, not exact numbers of downloads, were available. There were 20 ranges of downloads, and each was assigned a download level going from 1 (the smallest) to 20 (the highest). For the analysis the smallest value in each of the 20 ranges was also used as an alternative to the download levels. This was done to improve rigour of the analysis by using two approaches to estimate number of downloads from the available range of downloads.”

“Multiple linear regression (MLR) was used to model the relationship between app quality scores and the apps’ user ratings and downloads. R squared and adjusted R squared metrics were used to measure the fitness of the models. For all statistical tests, a p-value <.013 (Bonferroni-corrected for multiple hypothesis testing) was considered statistically significant. If there are any links among user ratings and downloads, and quality scores they should be revealed by spearman correlations and/or MLR.”

Results: Page 11, Lines 224-225: You may want to consider using a different method for differentiating between your scores on Figures 1 and 2 as these colours may be difficult for someone who is colour-blind to read.

Reviewer 2 response A3: We acknowledge that people with colour-blindness may not be able to differentiate the scores in the figures. Hence, in addition to using colours, we have now labelled figures 1 and 2 with text.
